# Increased Risk of Aging-Related Neurodegenerative Disease after Traumatic Brain Injury

**DOI:** 10.3390/biomedicines11041154

**Published:** 2023-04-11

**Authors:** Sarah Barker, Bindu D. Paul, Andrew A. Pieper

**Affiliations:** 1 Center for Brain Health Medicines, Harrington Discovery Institute, University Hospitals Cleveland Medical Center, Cleveland, OH 44106, USA; sxb878@case.edu; 2 Department of Psychiatry, Case Western Reserve University, Cleveland, OH 44106, USA; 3 Geriatric Psychiatry, GRECC, Louis Stokes Cleveland VA Medical Center, Cleveland, OH 44106, USA; 4 Institute for Transformative Molecular Medicine, School of Medicine, Case Western Reserve University, Cleveland, OH 44106, USA; 5 Department of Pathology, School of Medicine, Case Western Reserve University, Cleveland, OH 44106, USA; 6 Department of Pharmacology and Molecular Sciences, Johns Hopkins University School of Medicine, Baltimore, MD 21211, USA; bpaul8@jhmi.edu; 7 Department of Psychiatry and Behavioral Sciences, Johns Hopkins University School of Medicine, Baltimore, MD 21211, USA; 8 The Solomon H. Snyder Department of Neuroscience, Johns Hopkins University School of Medicine, Baltimore, MD 21211, USA; 9 Lieber Institute for Brain Development, Baltimore, MD 21205, USA; 10 Department of Neuroscience, School of Medicine, Case Western Reserve University, Cleveland, OH 44106, USA; 11 Translational Therapeutics Core, Cleveland Alzheimer’s Disease Research Center, Cleveland, OH 44106, USA

**Keywords:** traumatic brain injury, neurodegeneration, dementia, amyotrophic lateral sclerosis, frontotemporal dementia, Parkinson’s disease, Alzheimer’s disease, oxidative stress, proteostasis, neuroinflammation

## Abstract

Traumatic brain injury (TBI) survivors frequently suffer from chronically progressive complications, including significantly increased risk of developing aging-related neurodegenerative disease. As advances in neurocritical care increase the number of TBI survivors, the impact and awareness of this problem are growing. The mechanisms by which TBI increases the risk of developing aging-related neurodegenerative disease, however, are not completely understood. As a result, there are no protective treatments for patients. Here, we review the current literature surrounding the epidemiology and potential mechanistic relationships between brain injury and aging-related neurodegenerative disease. In addition to increasing the risk for developing all forms of dementia, the most prominent aging-related neurodegenerative conditions that are accelerated by TBI are amyotrophic lateral sclerosis (ALS), frontotemporal dementia (FTD), Parkinson’s disease (PD), and Alzheimer’s disease (AD), with ALS and FTD being the least well-established. Mechanistic links between TBI and all forms of dementia that are reviewed include oxidative stress, dysregulated proteostasis, and neuroinflammation. Disease-specific mechanistic links with TBI that are reviewed include TAR DNA binding protein 43 and motor cortex lesions in ALS and FTD; alpha-synuclein, dopaminergic cell death, and synergistic toxin exposure in PD; and brain insulin resistance, amyloid beta pathology, and tau pathology in AD. While compelling mechanistic links have been identified, significantly expanded investigation in the field is needed to develop therapies to protect TBI survivors from the increased risk of aging-related neurodegenerative disease.

## 1. Introduction

### 1.1. Scope of the Problem

Traumatic brain injury (TBI) has an annual incidence of almost 70 million people worldwide and is a major chronic public health problem [1]. Notably, the problem has grown in prevalence as advances in neurocritical care have reduced TBI mortality rates [2]. In the United States alone, 22% of people have sustained at least one TBI with loss of consciousness in their lifetime [3] and there are now approximately 5 million people living with TBI-related disabilities at an estimated annual cost of 80 billion USD [4,5].

TBI is often of multimodal etiology, including aspects of direct contact injury, acceleration/deceleration injury, and blast-wave injury. Regardless of the cause, however, all forms of TBI initiate a complex and unique disease process of primary and secondary injury. Primary injury results from mechanical tissue deformation, including contusion and rapid axonal and vessel shearing [6,7]. Secondary injury is caused by a multitude of interrelated chronic pathologic processes, including ischemia, excitotoxicity, metabolic dysfunction, blood–brain barrier (BBB) deterioration, oxidative stress, and neuroinflammation [7,8]. Over the long term, TBI results in chronic and progressive axonal demyelination and degeneration, leading to neuronal loss and varying degrees of neuropsychiatric impairment [7,9].

Importantly, TBI is also a significant environmental risk factor for developing aging-related neurodegenerative disease [10]. Epidemiologic evidence shows that TBI is associated with a significantly increased risk of developing all forms of dementia. Various studies and meta-analyses found odds ratios ranging from 1.25–1.63, indicating that individuals with a history of TBI have a significantly increased risk of developing dementia [11,12,13]. Indeed, it is estimated that TBI accounts for 5–15% of the attributable risk for dementia [14]. Specifically, TBI is associated with amyotrophic lateral sclerosis (ALS), frontotemporal dementia (FTD), Parkinson’s disease (PD), and Alzheimer’s disease (AD). Among these, ALS and FTD are the least well established as being linked to TBI, while the evidence linking TBI to PD and AD is quite robust. Here, we review the epidemiology and current understanding of the mechanistic links between these complex disease processes in both animal models and humans.

### 1.2. Challenges in Studying the Role of TBI in the Increased Risk of Aging-Related Neurodegenerative Disease

Numerous epidemiologic studies have assessed TBI as a risk factor for aging-related neurodegenerative disease. Some of these studies have reported conflicting results, which have been attributed to variability in study design, definition of the TBI exposure variable, and ethnicity and age of study subjects [15]. Another complicating factor is that human studies frequently rely on self-reported TBI, which is susceptible to recall bias, in cases that lack administrative data for TBI-related health care. In addition, earlier studies often used small sample sizes due to the low prevalence of clinically diagnosed TBI in the 19th century [16]. Furthermore, the number and/or severity of injuries has not always been monitored in clinical studies. Outcome measures have also not been kept uniform across studies, with utilization of a wide variety of approaches, such as International Classification of Diseases (ICD) codes, National Institute of Neurological and Communicative Disorders and Stroke/Alzheimer’s Disease and Related Disorders Association (NINCDS/ADRDA) AD criteria, Diagnostic and Statistical Manual of Mental Disorders (DSM) classification, and stratification by postmortem neuropathology. Furthermore, reverse causality has not been carefully assessed historically, which is problematic because many neurodegenerative diseases predispose individuals to falls that can result in TBI. Additionally, the duration of the neurodegenerative disease process before diagnosis is often unclear [15]. Importantly, a recent review found that studies with clearly defined TBI exposure variables and longer follow-up periods were more likely to find an association between TBI and dementia [17]. Despite these challenges, however, multiple meta-analyses of the published literature have consistently identified a prominent association between TBI and increased risk of developing aging-related neurodegenerative disease.

To investigate the mechanisms by which TBI increases the risk of aging-related neurodegenerative disease, researchers predominately rely on animal models. Both TBI and neurodegenerative disease are difficult to model in the laboratory, however, due to the heterogeneous nature of these conditions. With respect to TBI, there are four laboratory models of isolated TBI that have historically been most widely used: weight-drop impact acceleration injury [18], controlled cortical impact (CCI) injury [19], fluid percussion injury (FPI) [20,21], and blast injury [22].

In weight drop and CCI injuries, a direct insult is applied to the brain with a weight or rigid impactor. There are many versions of weight drop and CCI models, some of which require a craniotomy and cause cortical tissue loss, and others without craniotomy that cause skull fracture and are associated with high mortality [19]. The FPI model also involves craniotomy, with subsequent infliction of a fluid pressure pulse on the dura. Lastly, blast injuries typically use a compression driven shock tube to simulate exposure to explosive blast, which models some forms of battlefield injury [7,23].

These 4 distinct rodent models of TBI mimic different aspects of clinical TBI in people, with the first three modeling mechanical head trauma and the pure blast model entailing isolated exposure to a blast wave. To model the multimodal nature of most forms of human TBI, we recently characterized an additional rodent model of TBI that uses an overpressure chamber to deliver precise and reproducible aspects of global concussion, acceleration/deceleration injury, and early blast wave exposure without the need for surgery [24]. This model of multimodal TBI initiates acute axonal degeneration and BBB damage that persists chronically and progresses to neuronal cell death and behavioral alterations related to human neuropsychiatric impairment, with metabolomic changes in the blood that mimic changes seen in human TBI patients [25,26,27,28,29,30,31]. Thus, while there is no single laboratory model of TBI that recapitulates all of the disease processes in human TBI, multiple models are valued for their ability to reproduce various aspects of the human condition. 

As with TBI, modeling aging-related human neurodegenerative disease in the laboratory is problematic. Here, researchers typically utilize genetic rodent models. However, most cases of aging-related human neurodegenerative disease are sporadic (90–95% for ALS and FTD, 85% for PD, and 92–96% for AD) and not related to any known genetic cause [32,33,34]. Despite the challenges in the field, however, there is much effort to study this important problem with the tools available. 

## 2. Potential Mechanisms of TBI-Mediated Increased Risk of All Forms of Aging-Related Neurodegenerative Disease

While TBI accelerates the age of cognitive decline by approximately three years in people across all forms of dementia, no single reason has been determined for this effect [35]. Indeed, it has become increasingly appreciated that TBI and other neurodegenerative diseases share many pathologic mechanisms, and that several forms of chronic neurodegeneration display mixed and overlapping pathologies. For example, approximately half of patients with probable AD show degenerative pathologies common to other forms of neurodegenerative disease [36]. Likewise, many of the sequelae of TBI overlap with underlying pathology of multiple neurodegenerative diseases. Here, we review current knowledge on the contribution of three different TBI-derived pathological mechanisms that generally increase the risk of all forms of aging-related neurodegenerative disease: oxidative stress, dysregulated proteostasis, and neuroinflammation.

### 2.1. TBI-Induced Oxidative Stress and Increased Risk of All Forms of Aging-Related Neurodegenerative Disease

Although the human brain accounts for only 2% of total body weight, it voraciously consumes more than 20% of the body’s oxygen [37]. Due to this extraordinarily high rate of metabolism, the brain generates high levels of reactive oxygen species (ROS). Paradoxically, the brain is also among the most vulnerable organs to oxidative damage. This is predominantly due to its high polyunsaturated fatty acid content that is readily altered by ROS and its relatively poor oxidative stress response mechanisms [38]. These factors synergistically generate a multitude of toxic loss-of-function and gain-of-function events in key cellular processes [39,40,41]. The regulation and dysregulation of the antioxidant defense network in the brain, however, is less well characterized.

Particularly damaging ROS in the brain that lead to axonal degeneration and neuronal cell death include superoxide, hydrogen peroxide, hydroxyl radical, nitric oxide, and peroxynitrite. Not surprisingly, cells have evolved multiple levels of antioxidant defense. For example, natural antioxidants in the body that deactivate ROS into stable non-toxic molecules include superoxide dismutase (SOD), catalase, peroxidase, heme oxygenase, and biliverdin reductase, as well as lower molecular weight water- or lipid-soluble free-radical scavengers, such as glutathione (GSH), ascorbic acid, bilirubin, ergothioneine, and cysteine-derived thiols [42,43,44,45]. GSH, one of the most abundant antioxidants in the cell, plays central roles in antioxidant defense and is also a cofactor for several enzymes and important in synaptic transmission [46,47,48]. Similarly, ascorbic acid, also known as vitamin C, participates in a wide variety of neuroprotective signaling pathways [49]. In addition to these molecules, gaseous signaling molecules such as hydrogen sulfide modulate redox balance in the brain through multiple mechanisms [50,51,52,53,54,55]. All of the brain’s antioxidant systems are tightly regulated to provide optimal response for maintaining cellular integrity.

**Figure 1:**
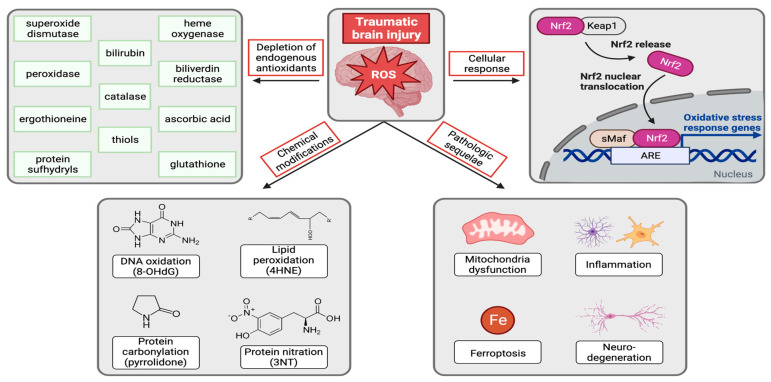
TBI leads to an increase in ROS, which chemically modify proteins, lipids, and nucleic acids, deplete endogenous antioxidants, and activate cellular responses via Nrf2. Oxidative stress causes mitochondrial dysfunction, inflammation, ferroptosis, and ultimately neurodegeneration.

A substantial body of evidence implicates oxidative stress across all forms of aging-related neurodegenerative disease, with lipids, proteins, and DNA all undergoing extensive oxidative damage (Figure 1) [40,42]. Furthermore, proteins that pathologically aggregate in neurodegeneration interact with redox-active metal ions and generate ROS. It is also well documented that TBI robustly generates ROS in both animal models and human patients. In animal models, there is evidence of protein nitration (3-nitrotyrosine: 3-NT), protein oxidation (carbonylation), lipid peroxidation (4-hydoxynonenal: 4-HNE), and DNA oxidation (8-hydroxy-2′-deoxyguanosine: 8-OHdG) within hours after TBI [56,57,58,59]. In human patients, 3-NT accumulates in the cerebrospinal fluid (CSF) and brain after TBI [60]. Importantly, the acute increase in ROS precedes chronic neuronal damage, suggesting a causative role [57,58].

In the brain, oxidative stress has also been linked to dysregulated iron homeostasis (Figure 1). For example, both TBI and aging-related neurodegenerative diseases induce changes in iron metabolism that increase iron deposition and elevate oxidative damage in the brain [61,62,63]. Ferroptosis, an iron-mediated form of cell death, has also been observed in TBI [64,65], and counteracting ferroptosis mitigates TBI symptoms [66]. Importantly, ferroptosis can be countered by several antioxidant proteins and metabolites, such as nuclear factor erythroid 2-related factor 2 (Nrf2) activation, GSH, and cysteine, the metabolism of which is frequently disrupted in neurodegenerative diseases, viral diseases, and aging [67]. 

Interestingly, increases in ROS are also associated with reduced antioxidant defense mechanisms (GSH, SOD, ascorbate, and protein sulfhydryls) after TBI in rodent models, consistent with the notion that antioxidant molecules and proteins counteract or prevent oxidative damage (Figure 1) [57,68]. In humans, TBI also exhausts the antioxidant reserve over the course of 7 days, as evidenced by reduced ascorbate and GSH [69]. 

One important regulatory mechanism of antioxidant defense in the brain is the Nrf2 transcription factor that plays a pivotal role in maintaining cellular redox balance in response to oxidative stress. Under basal conditions, Nrf2 is sequestered in the cytosol by Kelch-like ECH-associated-protein 1 (Keap1) and targeted for routine degradation [70]. In response to oxidative stress, however, Nrf2 dissociates from Keap1 and translocates to the nucleus, where it activates transcription of oxidative-stress-response genes (Figure 1) [71]. This phenomenon has been reported after TBI [72], and pharmacologic activation of Nrf2 rescues oxidative damage and neurologic dysfunction after TBI [73]. By contrast, Nrf2 knockout mice show more severe oxidative stress and neurologic dysfunction after TBI [73]. Nrf2 also links redox homeostasis to pain perception mechanisms and tolerance [74]. 

Overall, it is well-accepted that TBI increases ROS production and depletes antioxidant defense systems in the brain, leading to protein, lipid, and DNA damage that collectively impair neuronal function and eventually lead to neurodegeneration (Figure 1). Thus, TBI-induced oxidative stress represents a link to increased risk of developing all forms of aging-related neurodegenerative disease.

### 2.2. TBI-Induced Dysregulated Proteostasis and Increased Risk for All Forms of Aging-Related Neurodegenerative Disease

All neurodegenerative diseases share the common pathologic hallmark of aberrant protein aggregation, and there is evidence that TBI dysregulates key proteostatic processes that influence this system. These include the heat shock response, the unfolded protein response, the ubiquitin proteasome system, and autophagy.

Heat shock response is the process by which cellular stress, including elevated ROS, results in transcription-factor-driven expression of heat shock proteins (HSPs). HSPs are chaperones that refold proteins and prevent their aggregation by binding hydrophobic residues [75]. The heat shock response is induced after TBI in both rodent and human TBI [76,77,78,79]. Interestingly, genetically impairing the heat shock response by eliminating Hsp70 confers worse outcomes after TBI [80]. Conversely, genetic and pharmacologic activation of the heat shock response via Hsp70 is protective in rodent models of TBI [80,81].

The unfolded protein response upregulates protein-folding chaperones, reduces protein synthesis, degrades permanently misfolded proteins, and in cases of severe stress, initiates apoptosis. The unfolded protein response is coordinated by three arms: protein kinase RNA-like ER kinase (PERK), inositol-requiring enzyme 1 (IRE-1), and activating transcription factor 6 (ATF-6) in the endoplasmic reticulum (ER). Importantly, this system is induced in rodents after TBI [82,83], and its inhibition is protective [84,85,86].

The ubiquitin proteasome system, which selectively degrades misfolded and damaged proteins, is also dysregulated by TBI. For example, rodent TBI is associated with accumulation of ubiquitinated proteins in the brain, in conjunction with reduced protease activity [87,88]. The ubiquitin proteasome system is also dysregulated after TBI in human patients [89]. 

Lastly, accumulated misfolded proteins are also normally degraded by autophagy, through sequestration into autophagosomes that fuse with lysosomes for cargo degradation. Notably, autophagic flux is reduced after TBI in both animal models and human patients, and thus contributes to dysregulated proteostasis [89,90,91]. 

Overall, there is substantial evidence for widespread dysregulation of proteostasis after TBI. Given the commonality of impaired proteostasis across all forms of neurodegeneration, this is considered a general mechanistic link between TBI and aging-related neurodegenerative disease.

### 2.3. TBI-Induced Neuroinflammation and Increased Risk of All Forms of Aging-Related Neurodegenerative Disease

Neuroinflammation is another prominent component of aging-related neurodegenerative disease [92,93,94,95]. Typically, this process is indicated by reactive morphology of astrocytes and microglia, which secrete inflammatory mediators into the brain parenchyma [96]. Acutely after TBI, the immune response is crucial for clearing debris. However, chronic inflammation, which is seen years after injury in human patients, can perpetuate injury and contribute to aging-related neurodegenerative disease [97]. Recently, TBI has also been linked to development of senescence-associated phenotype (SASP)-derived inflammaging in the brain, which is a chronic low-grade sterile inflammation previously established to contribute to brain aging [98].

During the acute primary injury phase of TBI, there is a rapid release of damage-associated molecular signaling events that stimulate local and infiltrating immune cells to secrete toxic interleukin 6, interleukin 1 beta, and tumor necrosis factor [97]. Notably, blocking aspects of this process has been shown to be neuroprotective in an animal model of TBI [99]. TBI also induces astrogliosis, in which resident immune cells proliferate, change their morphology, and secrete cytokines and chemokines that potentiate neuroinflammation [100]. In addition, necrotic tissue releases double-stranded DNA, which activates the cyclic GMP-AMP synthase and stimulator of interferon genes (cGAS-STING) pathway. Here, cyclic GMP-AMP synthase produces cGAMP, which stimulates expression of pro-inflammatory interferon genes [101]. After TBI, excessive activation of the cGAS-STING pathway mediates neuroinflammation with elevated type-I interferons [102,103]. STING signaling is increased in both rodent and human TBI, and genetically ablating STING is neuroprotective in TBI [104,105]. 

The proinflammatory milieu induced by TBI also polarizes microglia into an M1-like state with reduced phagocytic activity and enhanced secretion of pro-inflammatory cytokines and free radicals. This toxic transition contributes to neurodegeneration. Conversely, microglia can also be neuroprotective when they are polarized to an M2-like state, in which they aggressively phagocytose debris, secrete anti-inflammatory cytokines and trophic factors, and augment postnatal neurogenesis. In rodent models, TBI induces a mixed M1- and M2-like phenotype in microglia, and much remains to be discovered about how this system could be manipulated to protect patients from TBI-induced acceleration of aging-related neurodegenerative disease [106,107,108]. 

## 3. Association of TBI with ALS and FTD

### 3.1. Epidemiology of TBI-Induced Increased Risk of ALS and FTD

Multiple studies have demonstrated an increased risk of ALS among retired professional athletes of contact sports such as football, soccer, and rugby [109,110,111]. While TBIs are certainly enriched in these individuals compared to the general population, these studies did not specifically account for history of TBI. An analysis of World War II veterans in 1980, however, found that, “men dying of ALS more often had a history of injury [TBI] 15 or more years before death than did the controls during the same period” [112]. Other studies have identified an increased risk for ALS in individuals who had a TBI within one [113] or ten [114] years of disease onset, but did not see an association at all time points. It is difficult to parse the directionality of this relationship because ALS is also associated with motor symptoms that predispose patients to falls. However, one study did not find any association between other physical injuries that could result from falls (trunk, arm, or leg) and ALS, suggesting specificity of the association with TBI [114]. Overall, a meta-analysis of studies published between 1980 and 2011 found insufficient evidence to support an association between a single TBI and ALS [115]. In addition, another study found that a history of TBI does not worsen disease progression or pathology in patients with ALS [116]. Thus, the association between TBI and ALS, while often discussed, remains unclear.

This relationship is perhaps further complicated by the evolution of understanding that ALS and FTD in some cases have overlapping pathology. FTD is a group of disorders associated with progressive damage to the temporal and frontal lobes of the brain associated with personality, language, and behavior, leading to impulsive behavior and communication difficulties. Although FTD is not usually associated with motor symptoms, upwards of 15% of people with FTD do experience motor neuron degeneration, termed FTD with ALS. In these cases, symptoms of FTD traditionally precede ALS. Approximately half of ALS patients also experience some degree of change in their thinking and behavior. Notably, three retrospective case control studies found a significantly increased risk for FTD in people who suffered from TBI [117,118,119]. More recently, it has been shown that a mutation in the noncoding region of the chromosome 9 open reading frame 72 (C9ORF72) locus, where an expansion of the hexanucleotide GGGGCC occurs, is not only a leading cause of ALS but also found in ~40% of cases of FTD [120,121]. Strikingly, prior TBI is specifically associated with advanced onset of disease in patients carrying this hexanucleotide repeat expansion [122]. Future well-controlled prospective studies will be required to elucidate the complex relationship between TBI and risk of developing ALS and FTD.

### 3.2. Potential Mechanisms of TBI-Induced Increased Risk of ALS and FTD

There is conflicting evidence of whether TBI accelerates or worsens disease in the most common animal model of ALS, which overexpresses a mutant form of human SOD1 (G93A) associated with a small number of human ALS patients. One group found that a single controlled cortical impact TBI did not affect disease onset or survival [123], but that repeated mild TBI caused earlier onset of motor symptoms, chronic motor deficits, and decreased cortical thickness in mutant SOD1 rodents [124,125]. However, another group found that a mild stab-wound injury to the motor cortex did not affect disease onset or progression in three different genetic rodent ALS models (SOD1, TAR DNA binding protein 43 (TDP-43), and fused in sarcoma (FUS)) [126]. It should be noted, however, that stab wounds to the head are one of the least frequent forms of human TBI and have not been specifically epidemiologically linked to aging-associated neurodegenerative disease. Lastly, another study showed that closed head cortical impact did not alter age of onset or lifespan in SOD1 mutant rodents but did worsen disease by more greatly impairing grip strength and increasing electromyography changes [127]. Thus, mechanistic studies show mixed evidence for an association between TBI and increased risk of ALS. However, as described below, substantial evidence indicates that TBI initiates related forms of TDP-43 and motor cortex pathology.

#### 3.2.1. TBI-Induced TDP-43 Pathology and ALS and FTD

A major proposed link by which TBI may increase the risk of ALS and FTD relates to TDP-43. TDP-43, a member of the TAR DNA-binding protein family, binds single-stranded DNA, RNA, and proteins. TDP-43 is implicated in mRNA stability, apoptosis, and cell division. However, its full array of physiologic functions in the brain is not yet fully understood. In both ALS and FTD, TDP-43 aberrantly localizes to the cytoplasm, where it aggregates and forms ubiquitin-positive inclusions [128]. In mouse models of TBI, axonal damage also increases TDP-43 expression, which then mislocalizes to the cytoplasm [129,130,131,132]. Interestingly, TDP-43 pathology after TBI may be transient, as TBI in three different ALS models (SOD1, TDP-43, FUS) acutely increases phosphorylated TDP-43 granules [126]. In human patients as well, a single TBI is sufficient to increase intraneuronal TDP-43, in the absence of the post-translational phosphorylation of TDP-43 that is typically associated with TDP-43 accumulation in ALS and FTD [133]. 

#### 3.2.2. TBI-Induced Motor Cortex Lesions and ALS and FTD

ALS involves death of motor neurons. In animal models, TBI causes progressive atrophy in the motor cortex and degeneration of the corticospinal tracts, resulting in muscle atrophy and motor deficits reminiscent of ALS [132]. ALS typically presents with focal motor symptoms, with onset in one or more myotomes spreading to adjacent muscles. A small study of 18 cases of sporadic ALS following frontotemporal cortical lesions (latency 8–42 years) found that the lesion site was frequently contralateral to the site of symptom onset, suggesting that TBI may be one “hit” that could lead to disease in conjunction with other genetic and non-genetic factors [134].

In summary, substantial challenges in disease definition and reliable animal models have precluded the firm establishment of whether and how TBI contributes to ALS and FTD. Substantial evidence, however, indicates that this topic is worthy of ongoing investigation.

## 4. TBI and PD

### 4.1. Epidemiology of TBI-Induced Increased Risk of PD

Many studies have assessed the relationship between TBI and PD, with a majority finding a significant association with odds ratios ranging from 0.6–6.2 [135]. Conversely, some studies did not find any association between TBI and PD [136,137]. Importantly, two meta-analyses of the published literature found a significant association between TBI and PD (Table 1) [138,139]. Interestingly, higher-severity TBI has been associated with higher risk of developing PD, further supporting a causative association [140,141,142].

It is important to recognize, however, that many epidemiologic studies investigating the association between TBI and PD are confounded by reverse causality, because PD is characterized by motor impairment, which can lead to TBI from falls. Overall, 60% of PD patients report at least one fall and 40% report recurrent falls, compared to 15% of the general older population [143]. While analysis of a large dataset from the Danish national hospital register found an odds ratio of 1.5 (95% confidence interval 1.4–1.7), this was almost completely driven by head injuries that occurred in the three months prior to PD diagnosis (odds ratio 8.0, 95% confidence interval 5.6–11.6) when study subjects were likely to have already experienced motor impairment. When only TBIs more than ten years prior to PD were considered, this study found no association [135]. In a study that controlled for trauma by incorporating non-TBI trauma controls (fractures) in patients over the age of 55, however, TBI patients were significantly more likely to be diagnosed with PD (hazard ratio 1.44) [141]. In summary, further prospective cohort studies are needed to elucidate the temporal relationship between TBI and PD.

### 4.2. Potential Mechanisms of TBI-Induced Increased Risk of PD

Evidence suggests that TBI initiates pathologic alpha (α)-synuclein loss of function and aggregation and directly harms dopaminergic neurons. Interaction between TBI and toxins known to cause PD provides further evidence for a mechanistic relationship. Further research in animal models that can recapitulate the full spectrum of PD pathology is needed to understand the mechanistic relationship between TBI and PD. 

#### 4.2.1. TBI-Induced α-Synuclein Pathology in PD

The α-synuclein protein composes ~1% of all cytoplasmic proteins in neurons and is found predominantly in presynaptic axon terminals where it interacts with phospholipids and proteins [144,145]. Interestingly, α-synuclein was originally identified as the principal non-amyloid beta component of amyloid deposits in AD plaques and blood vessels [146,147,148,149]. Subsequent work showed that α-synuclein also pathologically aggregates into insoluble fibrils that form Lewy body deposits in PD, as well as the related conditions of dementia with Lewy bodies and multiple system atrophy that are variably associated with TBI [150,151,152].

Levels of α-synuclein are elevated five- to ten-fold in cerebral spinal fluid after TBI in human patients [153,154]. In rodent models, TBI also causes α-synuclein accumulation with an age-dependent effect, most prominently in axons [155,156,157]. It has been hypothesized that TBI-induced oxidative and nitrosative stress causes conformationally modified forms of α-synuclein in both TBI and aging, which could contribute to PD [39]. Indeed, axonal α-synuclein pathology is one of the initial changes seen in PD [158]. Neuropathologic studies also show that severe TBI (loss of consciousness greater than 1 hour) is associated with increased accumulation of Lewy bodies in the substantia nigra and frontotemporal cortex. Importantly, this association remained even when only cases with a TBI before the age of 25 were included, controlling for reverse causality [159]. Furthermore, it is hypothesized that α-synuclein loses its function of regulating presynaptic neurotransmitter release in PD pathogenesis [160], and it has been shown that levels of monomeric alpha-synuclein are reduced regionally after TBI in a manner that correlates with synaptic loss [161]. Unfortunately, this topic is challenging to study in the laboratory because there are no TBI rodent models that on their own faithfully recapitulate the aggregated forms of α-synuclein observed in PD.

#### 4.2.2. TBI-Induced Dopaminergic Cell Loss in PD

Loss of dopaminergic neurons in the substantia nigra, which underlies akinesia, rigidity, and postural tremor in PD, is also thought to be a potential mechanism by which TBI increases the risk of PD. For example, dopamine transporter imaging revealed that human patients with TBI show a similar reduction in dopamine transporter levels in the caudate compared with PD [162]. Complementary to this, TBI in rodents leads to reduced expression of tyrosine hydroxylase and dopamine transporter in the substantia nigra [157], as well as reduced dopamine transporter in the cortex ipsilateral to the injury site [163]. Conversely, other studies found an increase in dopamine [164] and tyrosine hydroxylase [165] levels after TBI. Further research is needed to clarify the effect of TBI on dopaminergic cells. 

#### 4.2.3. Synergistic Effect of Toxins with TBI in PD

Environmental exposure to toxins such as pesticides, solvents, metals, and pollutants increase the risk of PD [146] and other forms of neurodegenerative disease [147,148]. Remarkably, TBI increases the vulnerability of rodents to toxins known to be associated with PD [149,150]. For example, while neither a low-dose toxin or mild TBI alone were damaging, the combination of both together caused accumulation of α-synuclein and loss of tyrosine hydroxylase positive cells in rats [150].

## 5. TBI and AD

### 5.1. Epidemiology of TBI-Induced Increased Risk of AD

Of all forms of aging-related neurodegenerative disease, the association between TBI and AD is the most well-established. This relationship was described as early as 1939 in various case studies that described AD-like clinical and pathologic presentations in patients after a single TBI [166,167]. Since then, many epidemiological studies have assessed the relationship between TBI and AD, often with varying results due to the methodologic challenges discussed above. Meta-analyses of the published literature revealed an approximately 50% increased risk of AD in TBI patients (Table 2). Although one published meta-analysis did not find an association between TBI and AD, the authors noted that it was not properly powered to assess this association [168]. Interestingly, two large meta-analyses found a male-specific effect [13,169].

In addition to increasing the risk of developing AD, TBI also accelerates its age of onset. For example, a population-based study found that time to develop AD was 8 years faster than expected in individuals with a history of TBI [171], and a recent study found that a single remote TBI accelerates the onset of AD by 3–4 years [172]. In addition, a history of TBI is also associated with greater amyloid beta deposition and more severe cortical thinning in AD patients [172]. 

### 5.2. Potential Mechanisms of TBI-Induced Increased Risk of AD

Although epidemiological and clinical studies have uncovered a clear link between TBI and an elevated risk of AD, the mechanisms underpinning this phenomenon are not completely understood. Studies assessing the effect of TBI in AD mouse models found that TBI worsens AD pathology. Most of these studies used 3xTg and APP/PS1 mouse models, which display amyloid beta and tau pathology, and the controlled cortical impact or weight-drop model of TBI. These studies found that TBI increased AD-like pathologies, including amyloid beta [173,174,175,176,177], phosphorylated tau [173,178], neuronal loss [174,179], and neuroinflammation [178,179,180]. Few studies performed cognitive behavioral tests, and those that did found cognitive deficits after TBI in AD models in contextual fear conditioning [181], radial-arm water maze [174], and Morris water maze [177,182]. Unfortunately, most studies have applied TBI at older ages (6–12 months) after AD pathology has developed in these models, and only analyzed at short timepoints (1–14 days). By contrast, there are only two published studies that applied TBI before the onset of severe pathology in AD models [129,181]. In this section, we review the molecular changes accompanying TBI that may result in developing AD, including TBI-induced brain insulin resistance, amyloid beta pathology, and tau pathology.

#### 5.2.1. TBI-Induced Brain Insulin Resistance in AD

Insulin, a peptide hormone secreted by the pancreas, crosses the BBB and engages insulin receptors in the brain. Indeed, emerging evidence shows that insulin plays an important role in brain metabolism, synaptic transmission, neuroinflammation, and vascular function. In addition, type 2 diabetes and central insulin resistance are known risk factors for AD [183]. Remarkably, there is growing evidence for pathologic insulin resistance in the brain in patients with AD, with increasing insulin resistance correlated with increased AD Braak stage [184]. Intranasal insulin administration also improves cognitive function in humans with mild cognitive impairment and AD [185,186].

Notably, TBI also induces brain insulin resistance in animal models [187] and human patients [188,189], which has been proposed to interfere with the ability of insulin to protect synapses against amyloid beta and tau oligomers [187,190]. Furthermore, brain insulin resistance in mice causes greater neurologic impairment after TBI [191]. In summary, TBI-induced insulin resistance may increase neuronal vulnerability to toxic amyloid beta and tau oligomers, increasing the risk of developing AD.

#### 5.2.2. TBI-Induced Amyloid Beta Pathology in AD

It is well documented, in both human patients and mouse models, that TBI induces amyloid plaques similar to AD [173,176,192,193]. In fact, amyloid plaques are found in approximately one-third of acute brain injuries [194]. Furthermore, long-term survivors of a single TBI have significantly more amyloid plaques than age-matched control subjects [193]. Surprisingly, decreased amyloid pathology has been reported after controlled cortical impact TBI in mice that carry an amyloid precursor protein mutation associated with early onset AD [195,196]. Additionally, a recent study found no significant difference in amyloid beta levels in Vietnam war veterans with or without a TBI [197]. Nonetheless, most of the literature reports an association of TBI with amyloid plaque accumulation in the brain.

A potential mechanism for this increase in amyloid beta is axonal injury caused by TBI. TBI, especially rotational injuries, can injure neurons through stretching tissue. Axonal injury disrupts the cytoskeleton and axonal transport, creating disconnected axon terminals. Here, amyloid precursor protein accumulates in the axons and can be cleaved into amyloid beta by presenilin-1 and beta secretase, which are also found in pathological axonal swellings [198,199]. It has been proposed that amyloid beta is released when damaged axons fully deteriorate, allowing formation of amyloid plaques [200,201].

There are also many studies showing that TBI acutely increases expression of amyloid precursor protein in injured axons in mice [202] and humans [203]. Additionally, caspases are activated after TBI [204]. For example, caspase 3 increases APP processing via disruption of beta secretase trafficking and degradation. Interestingly, TBI elevates beta secretase levels and activity [205]. Therefore, elevated beta secretase activity results in increased levels of pathogenic amyloid beta. Furthermore, caspase 3 also cleaves APP after TBI, generating more amyloid beta [206]. 

#### 5.2.3. TBI-Induced Tau Pathology in AD

Tau is a microtubule-binding protein essential for neuronal health, and abnormal accumulation of tau has been noted in axonal swellings in humans after TBI [31,32]. In pathologic states, including AD and TBI, tau undergoes post translational modifications (PTMs) that interfere with microtubule binding and increase its aberrant aggregation. These tau aggregates, termed neurofibrillary tangles (NFTs), are hyperphosphorylated and are a pathologic hallmark of AD. NFTs are found in approximately one-third of patients following moderate to severe TBI [193], and levels of brain phosphorylated tau correlate to TBI severity measured by the Glasgow Coma Scale [207]. 

In mice, TBI acutely increases levels of oligomeric and phosphorylated tau [208]. In the PS19 tauopathy mouse model, controlled cortical impact TBI also acutely increases phosphorylated tau pathology (AT8), which persists at least six months after injury [209]. Furthermore, in wild-type mice, TBI induces tau pathology that propagates and leads to synaptic loss and memory deficits [210]. While many studies have investigated tau PTMs after TBI in animal models (Table 3), these studies primarily rely on antibody-based PTM detection and focus primarily on phosphorylation. There is a need for a non-biased approach, such as mass spectrometry, to fully characterize the tau PTM landscape after TBI.

In addition to phosphorylation, tau is also acetylated in neurodegenerative disease. For example, AD brains show significant increases in tau acetylation at K274 and K281 [211,212]. In human brains, acetylated tau (ac-tau) is localized to thioflavin-s positive intracellular inclusions [213]. Ac-tau is also an early pathologic event in AD, beginning at the earliest Braak stages (one and two). Conversely, tau phosphorylation does not appear until Braak stages five and six [213,214,215]. Additionally, levels of ac-tau positively correlate with stage of disease and levels of tau accumulation, suggesting that tau acetylation is a driver of pathology [213]. The Gan group generated mice with acetylation mimics at two key pathologic residues, lysines 274 and 281. These mice, termed KQ mice, show significant tau pathology, impaired hippocampal synaptic plasticity, and memory deficits [216]. Notably, tau acetylation has also been linked to another risk factor for AD, female sex [217,218]. 

**Table 3:** Known tau PTMs induced by TBI in animal models.

PTM	Residue	Species	Model	Timepoint	Reference
Phosphorylation	S198	Rat	FPI	Acute	[219]
				Chronic	[219]
	S199	Rat	CCI	Acute	[220,221]
				Subacute	[220]
	S202	Mouse	CCI	Acute	[222,223,224,225]
				Chronic	[210,224]
			WD	Chronic	[226,227]
			Blast	Acute	[228]
				Subacute	[227,229,230]
		Rat	CCI	Acute	[220]
				Subacute	[220]
				Chronic	[231]
			FPI	Acute	[208,221]
				Subacute	[208,221]
				Chronic	[221,232]
	T205	Mouse	CCI	Acute	[222,223]
				Chronic	[210]
			WD	Chronic	[226]
			Blast	Acute	[228,233]
				Subacute	[229,230]
		Rat	CCI	Chronic	[231]
			FPI	Acute	[208,221]
				Subacute	[208,221]
				Chronic	[232]
	S212	Mouse	Blast	Acute	[229]
	S214	Mouse	WD	Chronic	[226,227]
			Blast	Acute	[229]
	T231	Mouse	CCI	Acute	[222,223]
				Chronic	[210]
			WD	Acute	[226,227]
				Subacute	[226,227]
				Chronic	[226,227]
			Blast	Acute	[227]
				Subacute	[8,9]
				Chronic	[227]
		Rat	WD	Acute	[234,235]
				Chronic	[235]
			FPI	Acute	[208,221]
				Subacute	[221]
				Chronic	[221]
	S262	Mouse	Blast	Acute	[228]
	S396	Mouse	CCI	Acute	[236]
				Chronic	[210]
			WD	Chronic	[227]
			Blast	Acute	[228,229]
				Subacute	[229]
		Rat	WD	Acute	[234,237]
			Blast	Acute	[237]
	S404	Mouse	CCI	Acute	[238]
				Chronic	[210,238]
			WD	Chronic	[227]
			Blast	Acute	[228]
	S416	Mouse	CCI	Acute	[222,223]
	S422	Mouse	CCI	Acute	[236]
Acetylation	K274	Mouse	CCI	Acute	[25]
				Subacute	[25]
			Blast	Acute	[25]
				Subacute	[25]
				Chronic	[25]
	K281	Mouse	CCI	Acute	[25]
				Subacute	[25]
			Blast	Acute	[25]
				Subacute	[25]
				Chronic	[25]

CCI = controlled cortical impact; WD = weight drop, FPI = fluid percussion injury, Blast = blast neurotrauma model, shock tube TBI, overpressure wave TBI, jet-flow overpressure multimodal TBI; Acute: <1 week; Subacute: 1 week–1 month; Chronic: >1 month.

We recently reported that tau is rapidly acetylated at these same sites after TBI as well, and that this acetylation occurs rapidly, persists chronically, and drives axonal degeneration [25]. Mechanistically, TBI induces GAPDH S-nitrosylation, which is the triggering pathological event that activates p300/CBP acetyltransferase to acetylate tau. S-nitrosylated GAPDH also inactivates SIRT1, one of the primary enzymes that removes acetyl groups from tau. Together, increased production and decreased clearance of ac-tau causes pathologic mislocalization of tau in the soma, which occurs following axonal initial segment degeneration, as established by the Rasband [239] and Gan laboratories [212]. This axon degeneration triggered by ac-tau also leads to neurodegeneration and cognitive impairment after TBI, and pharmacologically reducing ac-tau after TBI via multiple mechanisms rescued neurodegeneration and cognitive impairment [25]. Notably, ac-tau in the brain is more elevated in people with AD and a history of TBI, compared to people with AD and no history of TBI and healthy controls [25]. Therefore, ac-tau is an early and persisting pathologic event after TBI that may contribute to acceleration of AD. Importantly, blood levels of ac-tau directly correlate with brain levels, suggesting that ac-tau could be the first blood-based biomarker of neurodegeneration that directly reflects the abundance of a therapeutic target in the brain after TBI [25]. Furthermore, the rapid and persistent accumulation of ac-tau after TBI mimics the early disease state of AD, such that AD could be accelerated by TBI through this mechanism.

In summary, there is substantial evidence that TBI increases the risk and accelerates the onset of AD. Mechanistic studies suggest that multiple pathologic processes, including insulin resistance and pathology related to amyloid beta and tau, mediate this relationship. Further research should test whether therapeutically targeting these mechanisms will prevent the accelerated onset of AD after TBI in rodent models. 

## 6. Conclusions

Despite methodologic challenges, the current body of research overwhelmingly shows that TBI is associated with a significantly increased risk of age-related neurodegenerative disease. The National Institutes of Health 2022 triennial Alzheimer’s Disease and Related Dementias (ADRD) Summit to inform the national research agenda underscored this problem by including TBI [240]. Specifically, four priority areas were determined that together conceptualize TBI as a major contributor to dementia. These recommendations included (1) promoting interdisciplinary approaches to accelerate clinically meaningful research in this area; (2) characterizing clinical and biological phenotypes of neurodegenerative disease after TBI across diverse populations and histories of TBI, including validation of multimodal biomarkers; (3) establishing and strengthening infrastructure to support standardized methods with common data elements for antemortem and postmortem clinical and neuropathological characterization; and (4) extending basic and translational research to determine the mechanistic pathways and clinical manifestations of post-TBI neurodegenerative disease. 

**Figure 2:**
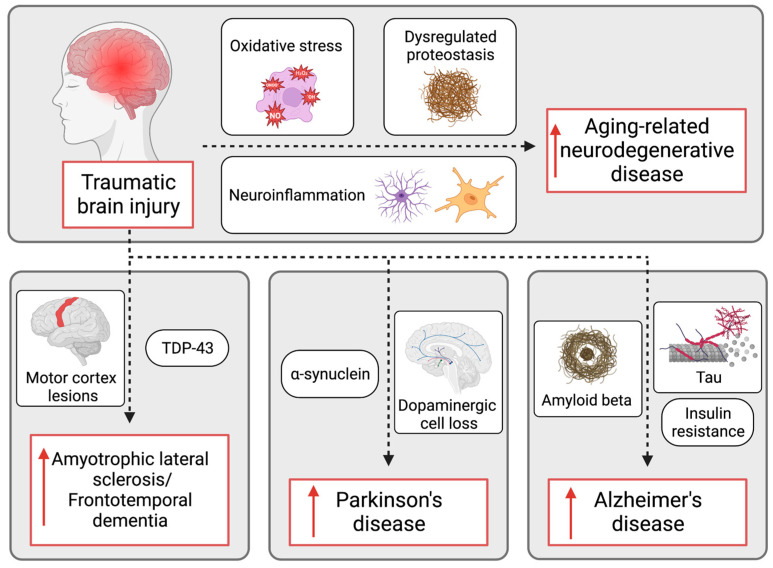
Potential mechanisms by which TBI increases the risk of aging-related neurodegenerative disease, both generally (dotted arrow in top panel) as well as specifically (dotted arrows in bottom panels) in ALS, FTD, PD, and AD. The red arrows indicate an increased risk of the specified condition.

It is well established that TBI initiates multiple pathologic processes that drive increased risk of neurodegenerative disease, including oxidative stress, impaired proteostasis, and both acute and chronic neuroinflammation, mechanisms that are common to varying degrees across all forms of neurodegenerative disease (Figure 2). Additional research testing whether therapeutically targeting these common pathologic changes after TBI will block the increased risk of aging-related neurodegenerative disease would greatly advance our knowledge of this problem and point towards potential neuroprotective therapies. Regarding specific forms of aging-related neurodegenerative disease, there is mixed evidence supporting the relationship between TBI and the increased risk of ALS and FTD. Further research should focus on large prospective epidemiology studies to assess this potential relationship. There is highly compelling evidence, however, that TBI increases the risk of developing PD and AD. 

With respect to PD, mechanistic studies provide evidence that TBI dysregulates α-synuclein, harms dopaminergic neurons, and synergizes with environmental toxins to accelerate the disease (Figure 2). Further research is needed to more rigorously establish potential mechanisms by which TBI increases the risk of PD, from which therapeutics can be designed. The largest body of research supports the association between TBI and AD. Preliminary mechanistic studies suggest that central insulin resistance may mediate this relationship, and there is also strong evidence that TBI initiates amyloid and tau pathology related to AD. Further research should investigate whether targeting these pathologic mechanisms initiated by TBI can prevent the increased risk and accelerated onset of AD in animal models. 

Overall, to understand the complex interplay between TBI and neurodegenerative disease, it is imperative for the field to generate and validate new animal models that combine TBI and aging-related neurodegenerative disease, in order to ultimately develop new neuroprotective therapies for patients. 

## Figures and Tables

**Table 1:** Meta-analyses assessing the association between TBI and PD.

First Author	Odds/Risk Ratio (*)
Jafari (2013) [138]	1.57 (1.35–1.83)
Balabandian (2023) [139]	1.48 (1.22–1.74)

* (95% confidence interval).

**Table 2:** Meta-analyses assessing the association between TBI and AD.

First Author	Odds Ratio (*)	Notes
Fleminger (2003) [169]	1.58 (1.21–2.06)	Male-specific effect
Perry (2016) [170]	1.40 (1.02–1.90)	Only mild TBI included
Li (2017) [13]	1.52 (1.26–1.80)	Male-specific effect

* Odds ratio (95% confidence interval).

## Data Availability

Not applicable.
